# Research Progress on Microwave Synthesis of 3d Transition Metal (Mn, Fe, Co, and Ni) Oxide Nanomaterials for Supercapacitors

**DOI:** 10.3390/molecules30081843

**Published:** 2025-04-19

**Authors:** Chengqi Sun, Maosheng Ge, Shuhuang Tan, Yichen Liu, Haowei Wang, Wenhao Jiang, Shoujun Zhang, Yin Sun

**Affiliations:** 1 Naval Architecture and Shipping College, Guangdong Ocean University, Zhanjiang 524088, China; sunchengqi@gdou.edu.cn (C.S.); gemaosheng@stu.gdou.edu.cn (M.G.); tanshuhuang@stu.gdou.edu.cn (S.T.); liuyichen@stu.gdou.edu.cn (Y.L.); whw1724016@stu.gdou.edu.cn (H.W.); 18256533425@stu.gdou.edu.cn (W.J.); 2 Guangdong Provincial Key Laboratory of Intelligent Equipment for South China Sea Marine Ranching, Guangdong Ocean University, Zhanjiang 524088, China

**Keywords:** microwave synthesis, 3d transition metal oxides, supercapacitors, electrode materials, electrochemical performance

## Abstract

3d transition metal oxides composed of Mn, Fe, Co, and Ni have emerged as promising candidates for supercapacitor electrode materials due to their high theoretical specific capacitance, abundant redox-active sites, variable oxidation states, environmental friendliness, and low cost. Various synthesis strategies have been developed to fabricate these nanostructures, including hydrothermal/solvothermal methods, sol–gel processing, and microwave-assisted synthesis. Among them, microwave irradiation technology, with its rapid heating characteristics and unique thermal/non-thermal effects, offers significant advantages in controlling crystallinity and particle size distribution, suppressing particle agglomeration, and enhancing material purity. Furthermore, microwave effects facilitate the self-assembly and morphological evolution of transition metal oxides, promote the formation of crystal defects, and strengthen interfacial interactions. These effects enable precise microstructural tuning, leading to an increased specific surface area and a higher density of active sites, ultimately enhancing specific capacitance, rate capability, and cycling stability. In recent years, microwave-assisted synthesis has made significant progress in constructing 3d transition metal oxides and their composites, particularly in the development of single-metal and binary-metal oxides, as well as their hybrids with carbon-based materials (e.g., graphene and carbon nanotubes) and other metal oxides. This review systematically summarizes the research progress on microwave-assisted techniques for 3d transition metal oxide-based nanomaterials, with a particular focus on the role of microwave effects in morphology control, interfacial optimization, and electrochemical performance enhancement. Additionally, key challenges in current research are critically analyzed, and potential optimization strategies are proposed. This review aims to provide new insights and perspectives for advancing microwave-assisted synthesis of 3d transition metal oxides in energy storage applications.

## 1. Introduction

**Figure 1:**
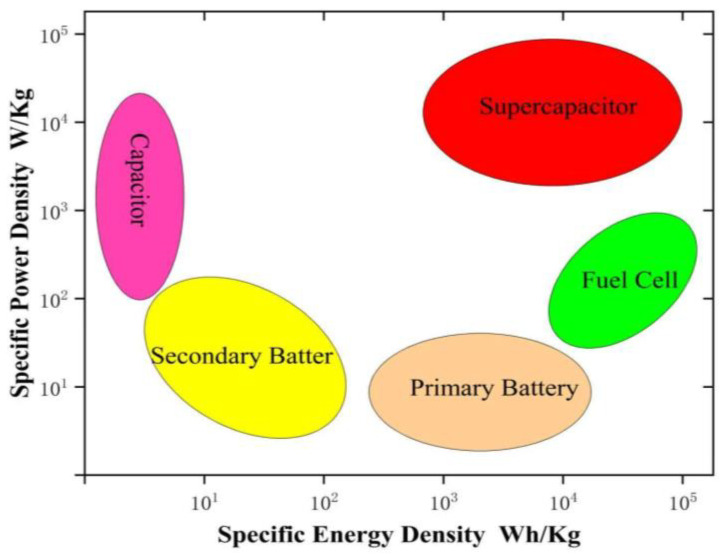
Ragone plot of common electrochemical energy storage systems.

The extensive use of traditional fossil fuels has led to massive greenhouse gas emissions, exacerbating global climate change while simultaneously causing environmental pollution and energy shortages. As a result, achieving the “dual-carbon goal” of carbon peaking and carbon neutrality has become a critical global initiative. In this context, the development and application of advanced energy storage devices are of paramount importance, as they can effectively mitigate the intermittency and instability associated with renewable energy utilization. Figure 1 presents a comparison of energy density and power density among various common energy storage devices. As illustrated, supercapacitors, as an emerging energy storage technology, exhibit several remarkable advantages, including ultra-high power density [1], an exceptionally long cycle life [2], a broad operational temperature range, high safety, and environmental friendliness. These superior characteristics make supercapacitors highly promising for applications in electric vehicles, power grid frequency regulation, and other energy storage systems.

Electrode materials play a pivotal role in determining the energy density, power density, and cycle life of supercapacitors. Currently, commonly used supercapacitor electrode materials include carbon-based materials, metal oxides, and conductive polymers. Among them, 3d transition metal oxides, such as MnO_2_, Fe_2_O_3_, Co_3_O_4_, NiMnO_3_, and MnCo_2_O_4_, have attracted significant attention due to their high theoretical specific capacitance, variable oxidation states, abundant redox-active sites, excellent cycling stability, and rapid ion transport capability. Moreover, these materials offer additional advantages, including environmental friendliness and low cost, making them highly promising candidates for next-generation supercapacitor applications.

**Figure 2:**
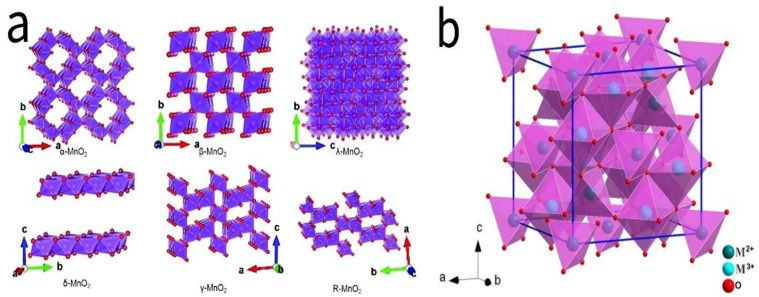
(**a**) Schematic crystal structures of crystallized manganese dioxide (α-, β-, γ-, δ-, λ-, and R-MnO_2_) and (**b**) crystal structure of cobalt tetroxide (Co_3_O_4_).

Figure 2 illustrates the crystal structures of several typical 3d transition metal oxides. These oxides exhibit diverse crystal structures, including layered, network, spinel, and perovskite structures. Such structural diversity endows these materials with highly tunable electronic properties, enabling effective modulation of charge transfer dynamics and electrochemical reactivity. Additionally, these structures provide an increased number of active sites, thereby optimizing electrochemical reactions. For instance, crystalline manganese dioxide (MnO_2_) exists in multiple polymorphs, including α-, β-, γ-, δ-, λ-, and R-MnO_2_ [3]. Specifically, α-MnO_2_ and γ-MnO_2_ feature large tunnel structures, which facilitate rapid electrolyte ion transport; δ-MnO_2_ possesses an open-layered structure with a relatively large interlayer spacing, promoting ion intercalation and deintercalation; and λ-MnO_2_ exhibits a network-like structure, where its high specific surface area not only increases the number of active sites but also further enhances ion transport efficiency.

Table 1 summarizes the commonly employed synthesis methods for 3d transition metal oxides along with their respective advantages and disadvantages [4]. As shown, electrochemical deposition, hydrothermal/solvothermal synthesis, sol-gel processing, liquid-phase co-precipitation, and microwave-assisted synthesis are widely utilized for fabricating 3d transition metal oxides. However, conventional solid-state or gas-phase reactions, as well as hydrothermal and solvothermal synthesis methods, often suffer from limitations in energy consumption, reaction efficiency, and product quality. In contrast, microwave-assisted synthesis has emerged as a promising approach for the fabrication of 3d transition metal oxides, owing to its advantages such as rapid processing, high efficiency, energy savings, and environmental friendliness.

**Figure 3:**
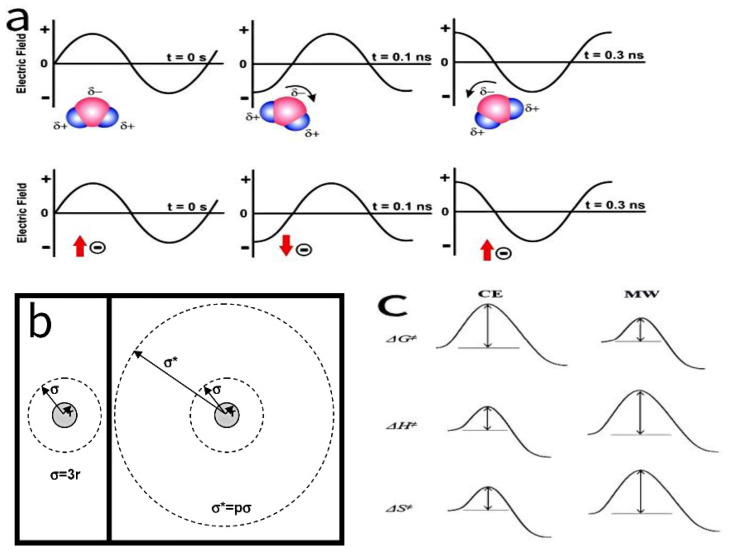
(**a**) Two primary heating mechanisms under microwave irradiation: dipolar polarization and ionic conduction. (**b**) Schematic illustration of non-interactive and interactive collision cross-sections. (**c**) Proposed relative changes in activation-free energy, enthalpy, and entropy associated with different synthesis methods (CE: conventional electric heating; MW: microwave heating).

Microwave synthesis technology, based on the principle of dielectric heating, rapidly and uniformly heats the reaction system through dipole polarization and ionic conduction (Figure 3a), enabling solvent molecules and polar precursors to quickly reach the required temperature [5]. In this process, microwave irradiation induces the formation of instantaneous dipole moments at the molecular and nanoscale levels, which enhances the dispersion forces between particles and introduces a steric hindrance factor (*p* > 1). Consequently, the “interactive collision cross-section (σ*)” of nanocrystals becomes significantly larger than the conventional “non-interactive collision cross-section (σ)” (Figure 3b), with the non-interactive collision cross-section assumed as σ = 3r, where r is the particle radius, thereby substantially increasing the effective collision frequency and promoting oriented attachment and orderly self-assembly [6]. Meanwhile, the effect of microwave irradiation increases the activation entropy (ΔS‡) of the reaction system, resulting in a reduction in the activation-free energy (ΔG‡), while the activation energy (Ea) and activation enthalpy (ΔH) are correspondingly increased (Figure 3c) [7]. This series of effects markedly enhances the nucleation rate, leading to the formation of numerous small, uniformly distributed nuclei and thus enabling precise control over crystallinity and particle size distribution. Moreover, due to the varying microwave absorption capacities of different reaction components, microwave heating can achieve precise local temperature control, effectively suppressing side reactions and particle agglomeration, ultimately significantly improving the material’s purity and reproducibility.

**Figure 4:**
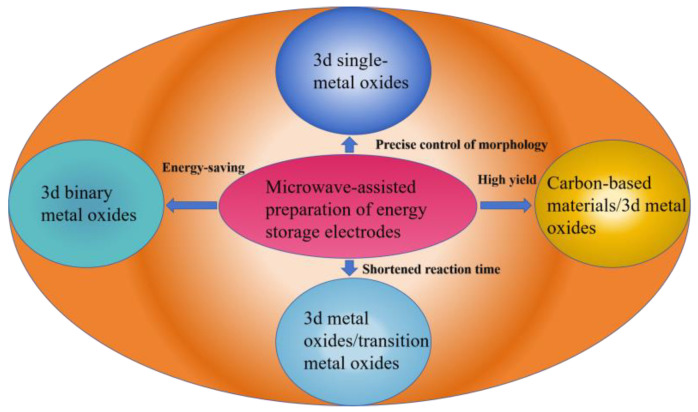
Research on the application of microwave-assisted synthesis of 3d transition metal oxides and their composites in energy storage electrodes.

Figure 4 illustrates the application of microwave-assisted synthesis of 3d transition metal oxides and their composites in energy storage electrodes. As shown in the figure, microwaves, as a form of non-ionizing electromagnetic radiation, possess strong penetration ability, allowing them to directly interact with the materials’ interiors and provide energy, resulting in uniform volumetric heating. Microwave heating is not only rapid and uniform, but its volumetric heating characteristics also significantly accelerate the nucleation process, reducing reaction times from hours to minutes while effectively lowering energy consumption. Furthermore, microwave heating helps to minimize the temperature gradient within the reaction system, promoting the rapid nucleation and growth of crystals, leading to high-yield, high-purity, and uniform products. More importantly, microwave-assisted synthesis enables precise control over the morphology of the products, offering a new strategy for the development of high-performance energy storage materials.

**Figure 5:**
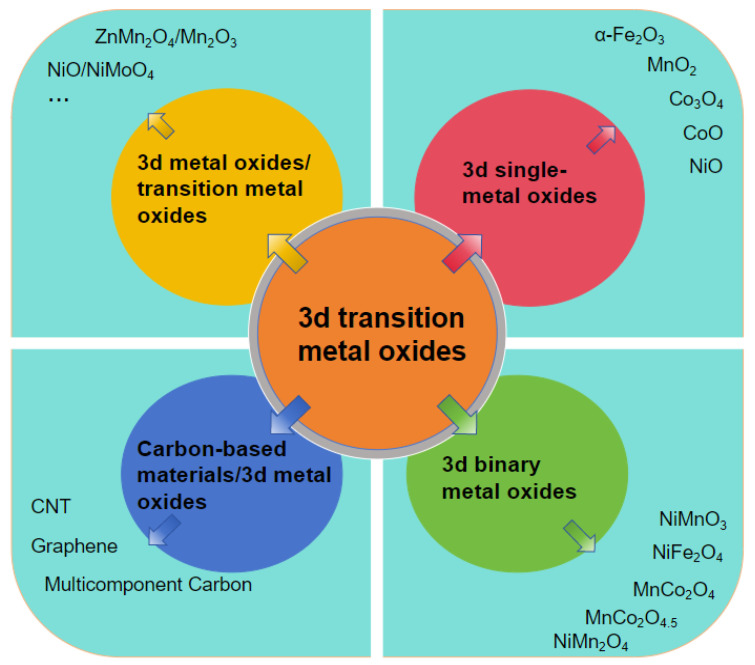
Schematic diagram of the framework structure for microwave-synthesized 3d transition metal oxide-based nanomaterials.

Figure 5 illustrates the schematic framework structure of the 3d transition metal oxide-based nanomaterials synthesized via microwave-assisted methods in this review.

This review systematically summarizes the research progress of microwave-assisted technology in the synthesis of 3d transition metal oxide-based nanomaterials, covering the fabrication and applications of single-metal oxides, binary-metal oxides, and their composites with carbon-based materials (e.g., graphene and carbon nanotubes) as well as other metal oxides. Owing to its rapid heating capability and unique thermal/non-thermal effects, microwave irradiation has demonstrated significant advantages in controlling crystallinity, regulating particle size distribution, suppressing particle agglomeration, and enhancing material purity. Furthermore, microwave-induced effects facilitate the self-assembly and morphological evolution of transition metal oxides, promote the formation of crystal defects, and strengthen interfacial interactions. These effects enable precise microstructural control, increase the specific surface area and the number of active sites, and ultimately lead to substantial improvements in specific capacitance, rate capability, and cycling stability. This review focuses on the mechanisms by which the microwave effect contributes to morphology control, interface optimization, and the enhancement of electrochemical performance. It also analyzes the key challenges in current research, such as the incomplete understanding of the reaction mechanisms underlying microwave effect regulation. Finally, the review proposes corresponding optimization strategies.

## 2. Results and Discussion

### 2.1. 3d Single-Metal Oxides

3d single-metal oxides composed of Mn, Fe, Co, and Ni have emerged as a research focus for supercapacitor electrode materials due to their multiple electron transfer reactions and tunable crystal structures. These materials not only offer abundant resources and cost-effectiveness, making them highly suitable for industrial applications, but also exhibit high theoretical specific capacitance (e.g., MnO_2_ up to 1370 F/g [8]) and numerous redox-active sites. Such properties enable them to overcome the limitations of conventional carbon-based materials in terms of specific capacitance and energy storage performance.

#### 2.1.1. Manganese-Based Oxides (MnO_2_)

Manganese-based oxides, known for their abundance and cost-effectiveness, are widely utilized as supercapacitor electrode materials due to their high theoretical specific capacitance, numerous reactive sites, and broad potential window [3]. Among them, MnO_2_ exhibits various crystal structures, including α-, β-, γ-, δ-, λ-, and R-MnO_2_, and provides an operational potential window of approximately 1 V in aqueous electrolytes, enabling high energy density [9]. Despite these advantages, MnO_2_ suffers from low intrinsic conductivity and limited surface-area utilization, resulting in a relatively low practical specific capacitance (typically around 250 F/g) [10,11]. Conventional MnO_2_ synthesis methods, such as thermal decomposition [12], electrodeposition [13], and sol–gel processing [14], often involve prolonged reaction times, high temperatures, and significant energy consumption, while also leading to the agglomeration of synthesized nanostructures. In contrast, microwave irradiation, with its rapid heating capability, facilitates the formation of unique microstructures, significantly reducing reaction times. Moreover, by precisely controlling the morphology of the synthesized material, microwave-assisted methods can effectively enhance the surface roughness and defect density of nanostructures, thereby further improving the electrochemical performance of MnO_2_.

**Figure 6:**
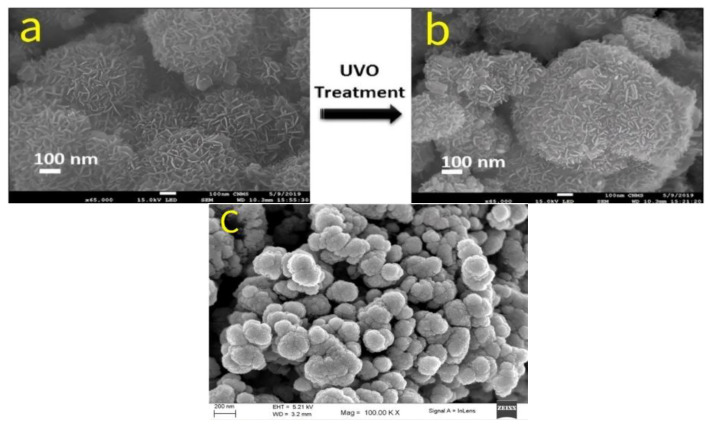
(**a**) TEM image of δ-MnO_2_. (**b**) TEM image of δ-MnO_2_-UVT. (**c**) FE-SEM image of pure δ-MnO_2_.

Samal et al. [15] successfully synthesized 2D δ-MnO_2_ electrode materials with a flower-like microsphere structure by integrating microwave irradiation technology with defect-engineering strategies. The study revealed that microwave irradiation, owing to its rapid heating capability, induces self-assembly of thin-layer nanosheets, resulting in the formation of a unique flower-like microsphere morphology (Figure 6a). After 30 min of microwave treatment, the 2D layered structure underwent deformation, with a smoother surface, more loosely bound nanosheets, and the formation of abundant vacancy structures (Figure 6b). This architecture not only provides additional active sites, facilitating ion transport, but also restricts excessive growth at the nanosheet edges, thereby optimizing the microstructure. Furthermore, Vimuna et al. [16] proposed a microwave-assisted synthesis method combined with simultaneous stirring, achieving the fabrication of porous MnO_2_ nanosheets in merely one-sixtieth of the synthesis time of the above-mentioned 2D δ-MnO_2_ electrode material, thereby significantly improving synthesis efficiency. Similarly, Raskar et al. [17] employed microwave-assisted co-precipitation to synthesize an alternative form of δ-MnO_2_ porous nanosheets by precisely tuning the nickel (Ni) doping concentration within the MnO_2_ lattice (Figure 6c). Under microwave irradiation, the nanosheet surface roughness increased significantly, accompanied by the formation of structural defects, leading to an enhanced specific surface area and a higher number of active sites, ultimately improving the energy storage performance of the material.

#### 2.1.2. Iron-Based Oxides (α-Fe_2_O_3_)

Iron-based oxides, with α-Fe_2_O_3_ as a typical representative, have shown broad application prospects in the field of electrochemical energy storage due to their high theoretical specific capacitance (α-Fe_2_O_3_: ~4800 F/g) [18], excellent structural stability [19], and non-toxic, environmentally friendly characteristics [20]. Microwave radiation technology can not only be used to synthesize α-Fe_2_O_3_ electrode materials with various nanostructures but also effectively induce the formation of internal defects in the material, such as oxygen vacancies.

**Figure 7:**
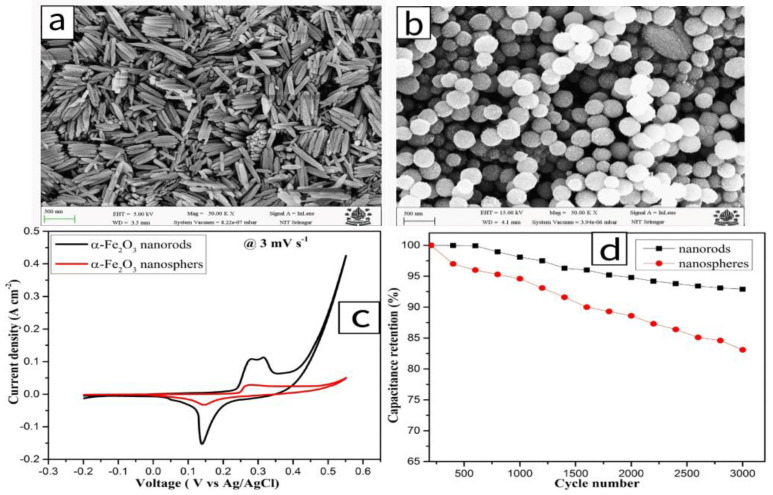
(**a**) FE-SEM image of α-Fe_2_O_3_ nanorods. (**b**) FE-SEM image of α-Fe_2_O_3_ nanospheres. (**c**) CV curves of nanorod and nanosphere electrodes at a scan rate of 3 mV/s. (**d**) Cyclic stability of nanorod and nanosphere electrodes at a scan rate of 10 mV/s.

Aalim et al. [21] successfully prepared porous α-Fe_2_O_3_ nanorods (Figure 7a) and α-Fe_2_O_3_ nanospheres (Figure 7b) rich in oxygen vacancies using rapid microwave radiation technology. Under microwave radiation, a large number of oxygen vacancies were formed inside the material, leading to improved storage capacitance. Electrochemical test results showed that in a 3 M KOH electrolyte, the α-Fe_2_O_3_ nanorods and nanospheres exhibited high specific capacitances of 1253 F/g and 715 F/g, respectively, at a scan rate of 3 mV/s (Figure 7c). Furthermore, after 3000 cycles, hematite nanorods maintained excellent cycling stability, with a capacitance retention rate of up to 93% (Figure 7d).

#### 2.1.3. Cobalt-Based Oxides (Co_3_O_4_ and CoO)

Cobalt-based oxides, including Co_2_O_3_, CoO, and Co_3_O_4_, are widely used as electrode materials for supercapacitors due to their high theoretical specific capacitance, significant reaction activity, and good electrochemical stability. They can efficiently participate in capacitive energy storage processes and exhibit excellent performance. Among them, Co_3_O_4_ possesses a high specific surface area (typically ranging from 80 to 150 m^2^/g, and even exceeding 200 m^2^/g in hierarchically porous or hollow structures) and stable physicochemical properties, and its surface and structure are easily tunable [22,23]. Its theoretical specific capacitance (3560 F/g) significantly surpasses that of MnO_2_ [24]. Meanwhile, CoO has an even higher theoretical capacitance of 4292 F/g [24], while also offering cost-effectiveness and environmental friendliness, making it a promising candidate for various applications. Among the various nanostructures, one-dimensional (1D) nanowires are favored for their high aspect ratio, minimal volume expansion and contraction, excellent three-dimensional diffusion properties, and strong volumetric capacitance kinetics [25]. Additionally, the directional microstructure of nanowires helps to form efficient ion transport paths, thereby enhancing ion diffusion rates and reducing energy loss. This makes them highly effective charge storage nanostructures for CoO and Co_3_O_4_ [26]. Microwave-assisted hydrothermal synthesis has shown unique advantages in the preparation of nanowire materials, as it can provide highly controllable reaction conditions at the microscopic scale, enabling the directional growth of CoO and Co_3_O_4_ nanowires. This approach allows for precise control over their morphology and crystal structure, improving material purity and significantly enhancing electrochemical performance. Furthermore, microwave-induced multivalent doping not only improves the specific capacitance of electrode materials but also broadens their operating voltage window, further optimizing the overall energy storage performance of the electrodes.

**Figure 8:**
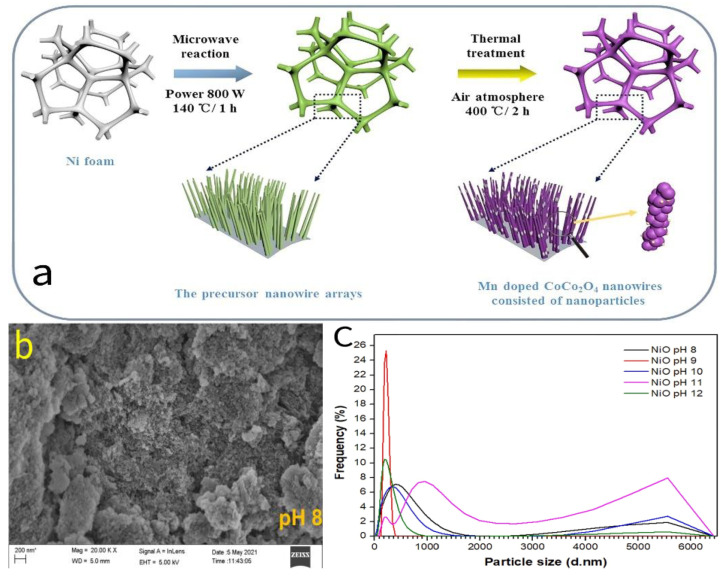
(**a**) Schematic of the synthesis process of Mn-doped CoCo_2_O_4_ porous nanowire arrays on Ni foam. (**b**) SEM image of NiO nanoparticles synthesized at pH 8 under a magnification of 20 K. (**c**) Particle size analysis of NiO nanoparticles synthesized at different pH values using Malvern.

Sun et al. [26] successfully synthesized 1D porous CoO nanowires through a microwave-assisted hydrothermal process (MHP) followed by post-heat treatment. Compared to traditional hydrothermal processes (CHPs) and solid-state reactions (SPs), the nanowire precursors synthesized using the MHP exhibited superior morphological characteristics. Specifically, the CoO precursor grew uniformly on the nickel substrate, with a smooth surface and a crosslinked needle-like morphology. After heat treatment, the initially smooth nanowires transformed into rough, porous structures formed by the accumulation of nanoparticles. Compared to the samples without microwave-assisted synthesis, the microwave-assisted prepared porous CoO nanowires exhibited enhanced specific capacitance (728.8 F/g at 1 A/g compared to 503.7 F/g) and improved cycling stability (82.3% retention after 5000 cycles at 15 A/g, compared to 76.5%). In addition, Sun et al. [27,28] further employed microwave-assisted hydrothermal synthesis to successfully fabricate porous CoCo_2_O_4_ nanowires on a nickel foam substrate, incorporating in situ isomorphic doping of Mn cations, as shown in the process pathway in Figure 8a. The study demonstrated that the unique effects of microwaves expanded the crystal parameters of the CoCo_2_O_4_ nanowires and significantly improved the material purity. Moreover, the expanded crystal parameters in the lattice defects provided favorable conditions for Mn doping. Using the microwave hydrothermal method, in situ doping of divalent Mn^2+^ and trivalent Mn^3+^ cations was successfully achieved, resulting in a substantial improvement in electrode performance. The specific capacitance increased from 875 F/g to 1389 F/g (at 1 A/g), the operating voltage window was extended to 1.1 V, and cycling stability was further enhanced. The overall improvement in electrode performance can be primarily attributed to microwave-induced multivalent Mn ion doping. The introduction of Mn^2+^ and Mn^3+^ facilitated the formation of reversible Mn^4+^, thereby providing additional redox reaction activity and enhancing the electrochemical storage capacitance. Additionally, this doping mechanism effectively suppressed the water splitting process, thus expanding the operating voltage window.

#### 2.1.4. Nickel-Based Oxides (NiO)

As a typical representative of nickel-based oxides, NiO is a p-type semiconductor that has attracted widespread attention in the field of supercapacitors due to its high theoretical specific capacitance (2573 F/g), excellent cycling stability, and strong redox properties [29,30]. Microwave radiation technology provides an efficient and controllable method for the synthesis of NiO materials, enabling precise regulation of crystallinity, particle size distribution, and microstructure. Specifically, microwave radiation promotes uniform particle distribution and effectively suppresses agglomeration [31,32].

Kumar et al. [33] used microwave radiation technology to precisely control the mesoporous structure of NiO during dynamic crystallization, successfully synthesizing NiO materials with a unique nanospherical plate-like morphology [34]. Further studies revealed that the synergistic effect of microwave parameters and the synthetic chemical environment plays a crucial role in the materials’ microstructural characteristics. For example, Salleh et al. [35] employed a microwave-assisted sol–gel method to synthesize NiO nanoparticles with different microstructural features under varying pH conditions. The study found that under the effective control of microwave radiation, when the pH value was 8, the resulting NiO nanoparticles exhibited the best crystallinity, a uniform particle size distribution (Figure 8c), and a regular spherical morphology, with significantly reduced agglomeration (Figure 8b).

### 2.2. 3d Binary Transition Metal Oxides

3d binary transition metal oxides (BTMOs) exhibit multiple oxidation states, enabling efficient multi-step redox reactions. They also possess a lower electron transfer activation energy and a higher electrical conductivity, which contribute to enhanced electrochemical performance. Furthermore, the synergistic interactions between multivalent metal ions in BTMOs, coupled with the multi-electron pair coordination effect facilitated by their unique structures, further improve their charge storage capabilities and charge–discharge efficiency. These advantages make BTMOs superior to single-component metal oxides in terms of capacitance and electrochemical activity, offering promising material candidates for the development of high-performance supercapacitors.

#### 2.2.1. Nickel–Manganese Composite Oxides (NiMn_2_O_4_ and NiMnO_3_)

On the one hand, nickel–manganese composite oxides exhibit a higher theoretical specific capacitance and abundant electroactive sites; on the other hand, these materials are non-toxic, cost-effective, and naturally abundant, making them highly advantageous for practical applications in electrochemical energy storage [36]. In binary nickel–manganese composite oxide electrodes, nickel and manganese elements participate synergistically in redox reactions. As the number of electron transfers increases, the electrical conductivity and theoretical specific capacitance of the material are significantly enhanced [37,38,39,40]. Among these materials, NiMnO_3_ with an ilmenite structure demonstrates excellent magnetic properties, electrochemical performance, and catalytic activity, achieving a high theoretical specific capacitance of 2168 F/g [41] along with outstanding cycling stability. Meanwhile, NiMn_2_O_4_ with a spinel structure exhibits superior electrical conductivity during redox reactions, with a theoretical specific capacitance of up to 2992 F/g [41], further highlighting its potential for energy storage applications. Studies have shown that microwave irradiation temperature significantly influences the microstructure and electrochemical performance of the final product. The unique mechanisms of microwave-assisted synthesis facilitate morphological evolution, further optimizing material properties. Therefore, the microwave-assisted hydrothermal method demonstrates distinct advantages in the fabrication of NiMn_2_O_4_ and NiMnO_3_ electrode materials, providing an effective strategy for the development of high-performance energy storage materials.

Du et al. [42] employed a microwave-assisted hydrothermal method combined with annealing at different temperatures (ranging from 350 °C to 650 °C) to grow NiMn_2_O_4_ electrode materials on 3D nickel foam in situ. They systematically investigated the effects of microwave irradiation temperature on the microstructure and electrochemical performance of the material. The results revealed that microwave irradiation temperature plays a crucial role in determining the morphology and electrochemical properties of the final product, with excessively low or high temperatures proving detrimental to material optimization. Under microwave irradiation, NiMn_2_O_4_ synthesized at 450 °C exhibited significantly optimized morphological features, characterized by a relatively large grain size, a high specific surface area, and well-defined porous structures, which collectively contributed to its superior electrochemical performance.

**Figure 9:**
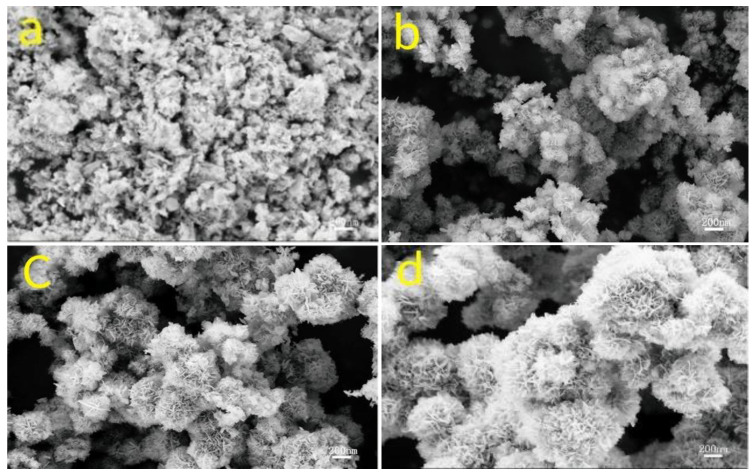
SEM images of flower-like NiMnO_3_ nanospheres synthesized at different microwave-assisted hydrothermal deposition temperatures: (**a**) 120 °C, (**b**) 140 °C, (**c**) 160 °C, and (**d**) 180 °C.

Compared to conventional hydrothermal methods, the unique effect of microwave irradiation temperature facilitates the formation of distinct morphological characteristics. For instance, Qiao et al. [43] successfully synthesized 3D nanoflower-like NiMnO_3_ electrode materials via a rapid and straightforward microwave-assisted hydrothermal approach. Their study demonstrated that the distinctive microwave effect significantly accelerated the morphological evolution process. As the microwave irradiation temperature increased, the microstructure of the material transitioned from a bulk-like morphology to a flower-like structure and ultimately grew further (Figure 9a–d), a phenomenon not observed in traditional hydrothermal synthesis. This unique hierarchical microsphere architecture provided abundant active sites and effectively facilitated rapid electron and ion transport, thereby greatly enhancing the electrochemical performance of the material.

Similarly, Sun et al. [44] successfully synthesized NiMn_2_O_4_ composed of flower-like microspheres self-assembled from nanosheets by introducing microwave assistance during the hydrothermal process on a 3D nickel foam substrate. SEM images revealed that under microwave irradiation at 160 °C, the resulting product exhibited a well-defined hierarchical flower-like microsphere morphology. The study indicated that the “microwave superheating effect” led to localized temperatures exceeding the target temperature over extended periods, thereby promoting the gradual aggregation of primary nanosheets into small microspheres, which subsequently grew into fully developed flower-like microsphere structures.

#### 2.2.2. Nickel–Iron Composite Oxides (NiFe_2_O_4_)

NiFe_2_O_4_, formed by doping Ni^2+^ into a Fe^3+^-dominated structure, exhibits a unique spatial arrangement of octahedral and tetrahedral sites, imparting the material with distinct electrical and magnetic properties. Moreover, NiFe_2_O_4_ is abundant in resources and exhibits good biocompatibility, thereby demonstrating excellent applicability in various applications [45,46,47]. In conventional synthesis methods, the fabrication of NiFe_2_O_4_ nanoparticles typically requires high-temperature calcination to achieve proper crystallization and morphology control. In contrast, the microwave-assisted combustion method offers a low-temperature and highly controllable alternative, enabling the efficient synthesis of NiFe_2_O_4_ nanoparticles without the need for high-temperature treatment. This method not only facilitates precise regulation of particle size and morphology, but also results in a high specific surface area, providing an efficient and feasible approach for NiFe_2_O_4_ nanoparticle preparation. For example, Kumar et al. [48] successfully synthesized NiFe_2_O_4_ nanoparticles using an eco-friendly microwave-assisted combustion technique, employing citrus lemon fruit extract as a natural fuel. Through microwave irradiation-induced self-combustion, the nanoparticles were synthesized without requiring high-temperature calcination. This approach resulted in nanoparticles with a reduced size and increased specific surface area, thereby significantly enhancing the contact area between the electrode and electrolyte. Consequently, ion adsorption and diffusion were promoted, improving the double-layer capacitance and further enhancing the materials’ overall energy storage performance.

#### 2.2.3. Manganese–Cobalt Composite Oxides (MnCo_2_O_4_ and MnCo_2_O_4.5_)

Similar to nickel–manganese composite oxides, manganese–cobalt composite oxides, including MnCo_2_O_4_ [49], CoMn_2_O_4_ [50], and MnCo_2_O_4.5_ [51], have attracted significant attention in the field of energy storage due to their high theoretical capacitance and abundant active sites. Additionally, these materials further enhance electrochemical performance through the synergistic effect of multivalent metal ions, while offering advantages such as high availability and ease of synthesis. These characteristics make manganese–cobalt composite oxides highly promising as battery-type electrode materials for supercapacitors [52]. Among them, MnCo_2_O_4_ demonstrates an exceptionally high theoretical capacitance of up to 3619 F/g, combining the superior electronic transport capability of manganese with the high oxidation potential of cobalt. Consequently, it exhibits excellent rate capability and capacitive behavior, making it highly efficient for energy storage applications [52,53,54,55,56]. Meanwhile, MnCo_2_O_4.5_ electrode materials have also been extensively investigated for energy storage applications due to their rich oxidation states and synergistic interactions between metal cations [57,58]. Studies have shown that nanostructured thin sheets with high specific surface areas can effectively enhance capacitance performance. By optimizing microwave processing parameters, MnCo_2_O_4_ and MnCo_2_O_4.5_ nanosheets with more uniform morphologies and superior electrochemical properties can be synthesized. Moreover, microwave-assisted synthesis can induce the formation of intrinsic structural defects, such as oxygen vacancies, further improving the electrochemical performance of these materials.

Krishnan et al. [59] were the first to employ microwave-assisted synthesis to achieve the rapid and scalable preparation of MnCo_2_O_4_ nanosheets. By precisely controlling the heating rate, pressure, and temperature during the microwave synthesis process, they were able to effectively control the uniform nucleation and growth of the material, resulting in a uniform particle distribution and an optimized microstructure.

**Figure 10:**
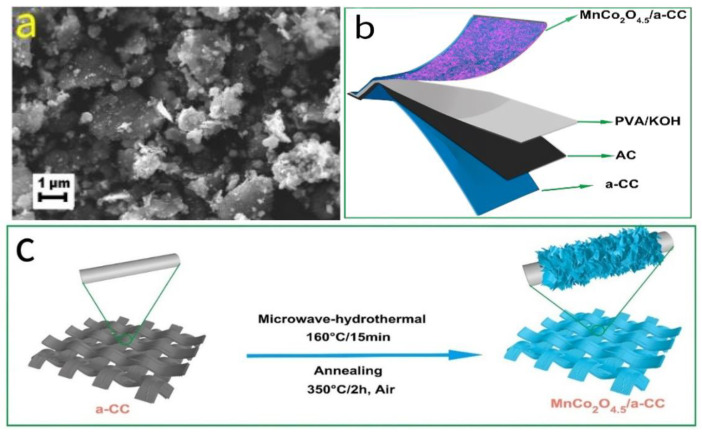
(**a**) SEM image of MnCo_2_O_4_ nanocubes prepared by the microwave method. (**b**) Schematic diagram of the designed all-solid-state asymmetric supercapacitor. (**c**) Schematic diagram of the preparation of MnCo_2_O_4.5_ porous nanosheets on carbon cloth.

Similarly, Sannasi et al. [60,61] utilized microwave-assisted synthesis with the addition of phenolphthalein as an organic indicator and successfully prepared MnCo_2_O_4_ nanosheets and MnCo_2_O_4_ nanocubes (composed of sponge-like aggregated nanosheets; see Figure 10a). For comparison, they also synthesized MnCo_2_O_4_ nanoparticles using a phenolphthalein-assisted hydrothermal method without microwaves. The results showed that, compared to conventional hydrothermal methods, the microwave-assisted synthesis of MnCo_2_O_4_ exhibited significant morphological differences, with larger microcrystal sizes and a more uniform distribution. Moreover, the MnCo_2_O_4_ nanosheets and MnCo_2_O_4_ nanocubes delivered specific capacitances of 802 F/g and 1053.5 F/g, respectively, at a current density of 1 A/g, significantly outperforming the MnCo_2_O_4_ nanoparticles prepared by conventional hydrothermal methods, further validating the effectiveness of microwave-assisted techniques in optimizing material structure and enhancing energy storage performance.

Moreover, the microwave-assisted synthesis method can effectively induce the formation of defects, making it an essential technique for the preparation of non-stoichiometric MnCo_2_O_4.5_. Sun et al. [62] proposed a time-efficient microwave-assisted hydrothermal method, successfully synthesizing highly crystalline MnCo_2_O_4.5_ nanosheets directly grown on carbon cloth (a-CC) without the use of binders. The synthesis process is illustrated in Figure 10c. The microwave-assisted approach facilitated the formation of a unique porous nanosheet structure in MnCo_2_O_4.5_ and induced the generation of abundant oxygen vacancies. These structural features not only provided rich porosity, increasing the material’s specific surface area, but also effectively enhanced electron transport, thereby improving its conductivity. Thanks to the microwave effect, the potential window of this material was significantly broadened to 1.05 V, much higher than those of previously reported MnCo_2_O_4.5_-based electrode materials (usually below 0.75 V). This result indicates that microwave-assisted synthesis not only enhances the capacitance (C) of the material but also significantly widens its working potential window (V), thereby optimizing the overall energy storage performance. The all-solid-state asymmetric supercapacitor assembled with MnCo_2_O_4.5_/a-CC//AC (design shown in Figure 10b) exhibited an ultra-wide potential window of 2.05 V, achieving a high energy density of 30.8 Wh/kg at a power density of 1025 W/kg, while also demonstrating excellent long-term durability and mechanical flexibility.

### 2.3. Carbon-Based Materials/3d Transition Metal Oxide Composite Materials

Carbon-based materials (such as graphene and carbon nanotubes) play a critical role in electrode materials. They not only enhance the adhesion between carbon and inorganic materials but also reduce charge transfer resistance. Additionally, by constructing buffer layers, they effectively suppress the volumetric expansion of the composite materials [63,64,65]. Furthermore, the high conductivity of carbon materials contributes to improving the charge transfer efficiency within the electrode, while providing mechanical support to transition metal oxides, thereby mitigating structural degradation and significantly enhancing cycle stability [66].

#### 2.3.1. Graphene-Based 3d Transition Metal Oxide Electrode Materials

Graphene is a two-dimensional monolayer material composed of sp^2^ hybridized carbon atoms, characterized by a honeycomb lattice structure [67,68]. Its excellent conductivity, thermal conductivity, chemical stability, and high specific surface area (theoretical value up to 2630 m^2^/g) make it an ideal electrode material [69,70,71]. When graphene is combined with 3d transition metal oxides, it provides abundant active sites, facilitating the adsorption and diffusion of electrolyte ions while constructing a three-dimensional conductive network that significantly accelerates electron transport. This composite material not only enhances electrochemical activity but also optimizes ion transport efficiency and improves mechanical stability. It possesses a high porosity and a large surface area, further boosting energy storage performance. In dry microwave radiation processing, by precisely controlling the microwave power and processing time, the microstructure of graphene/3d transition metal oxide composites can be effectively optimized, significantly enhancing interfacial interactions.

**Figure 11:**
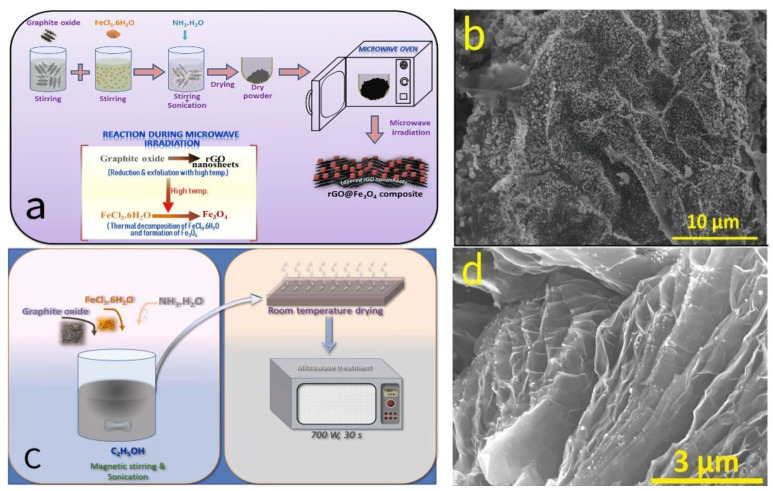
(**a**,**c**) Schematic illustrations of the synthesis of two different rGO@Fe_3_O_4_ nanocomposites. (**b**) FE-SEM and (**d**) SEM images of the rGO@Fe_3_O_4_ nanocomposites.

Kumar et al. [72,73] employed an efficient and simple dry microwave radiation synthesis method to successfully prepare two different morphologies of rGO@Fe_3_O_4_ nanocomposites, with the synthesis schematics shown in Figure 11a,c. By adjusting the microwave power and processing time, they achieved precise control over the microstructure of the graphene-based Fe_3_O_4_ composites. When the microwave power was set to 900 W and the processing time was 45 s, the resulting material exhibited polyhedral Fe_3_O_4_ nanoparticles evenly loaded on the surface of rGO (Figure 11b). In contrast, when the microwave power was reduced to 700 W and the processing time was shortened to 30 s, the Fe_3_O_4_ nanoparticles were nearly spherical and uniformly attached to the surface and edges of the rGO nanosheets (Figure 11d).

In addition, Kumar et al. [74] used the dry microwave strategy to successfully synthesize N-doped reduced graphene oxide nanosheets (N-rGO NSs) uniformly dispersed with CoO nanocrystals (N-rGO@CoOs). Under the rapid and uniform heating effect of microwave radiation, the surface activity of graphene was effectively activated, promoting the formation of defects and the introduction of oxygen-containing functional groups on the N-rGO NSs’ surfaces, thereby enhancing their chemical reactivity. Moreover, the microwave-induced local thermal effects helped achieve a uniform dispersion of the CoO precursor on the graphene surface, followed by in situ growth, forming a tight interface bonding. The composite structure of N-rGO@CoO not only effectively increased the material’s specific surface area and porosity but also significantly enhanced its electrochemical performance. At a scanning rate of 5 mV/s, the N-rGO@CoO electrode exhibited a high specific capacitance of 744.1 F/g, and after 5000 cycles, the capacity retention reached 91.3%, demonstrating excellent cycling stability.

#### 2.3.2. Carbon Nanotube-Based 3d Transition Metal Oxide Electrode Materials

Carbon nanotubes (CNTs), with their high conductivity, high aspect ratio, excellent electrolyte accessibility, and mesoporous structure, have been widely applied in supercapacitor electrode materials [75,76,77]. Leveraging these advantages, carbon nanotube-based 3d transition metal oxide electrode materials combine the high conductivity of CNTs with the high specific capacitance of metal oxides, exhibiting outstanding overall performance. These composite materials not only possess a high specific surface area but also exhibit excellent mechanical stability and efficient ion transport capabilities, making them highly promising in the field of supercapacitor energy storage. Electrode materials based on carbon nanotubes and 3d transition metal oxides, synthesized using microwave processes, significantly enhance the specific surface area and number of active sites by introducing nanoscale defects on the CNTs’ surfaces, thereby improving their electrochemical performance. Moreover, the “hotspot” effect induced by microwave irradiation accelerates the nucleation of metal ions and promotes the formation of layered heterostructures, further optimizing the materials’ microstructure and achieving superior energy storage performance.

**Figure 12:**
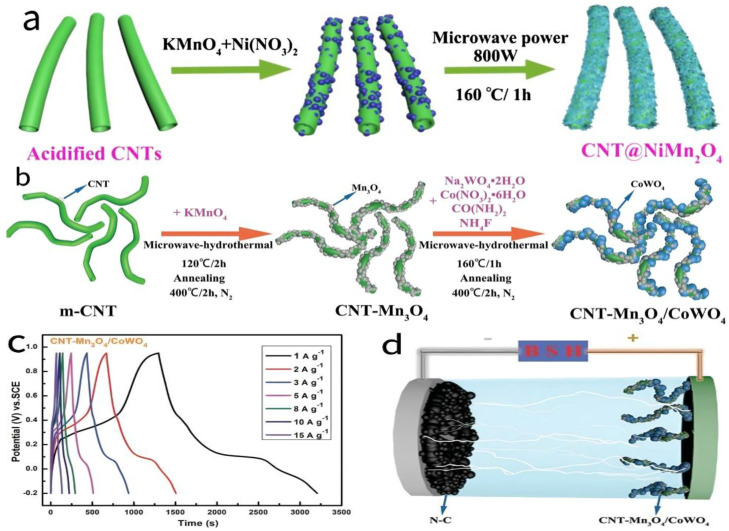
(**a**) Schematic illustration of the formation process of CNT@NiMn_2_O_4_ nanocomposites. (**b**) Schematic illustration of the synthesis process of CNT-Mn_3_O_4_/CoWO_4_ nanocomposites. (**c**) GCD curves of CNT-Mn_3_O_4_/CoWO_4_ at current densities ranging from 1 to 15 A/g. (**d**) Schematic diagram of the assembly principle of the CNT-Mn_3_O_4_/CoWO_4_//N-C BSH device.

Sun et al. [78] successfully synthesized CNT@NiMn_2_O_4_ core–shell nanocomposites via a microwave-assisted hydrothermal method, as shown in Figure 12a. Electrochemical tests revealed that the material achieved a specific capacitance of 915.6 F/g at a current density of 1 A/g, demonstrating excellent energy storage performance. This outstanding performance is mainly attributed to the synergistic effect between the CNTs and NiMn_2_O_4_ in the core–shell heterostructure, as well as the additional defects induced by microwave irradiation and acid pre-treatment. These structural characteristics effectively enhance the material’s specific surface area and the number of active sites, significantly improving its electrochemical performance.

Furthermore, Park et al. [79] utilized diethylene glycol (DEG) as a solvent to synthesize Fe_3_O_4_/CNT composite electrode materials through a microwave solvothermal process. Under microwave irradiation, metal ions preferentially nucleate in localized high-energy regions (“hotspots”), resulting in the uniform and ordered growth of Fe_3_O_4_ nanoparticles on CNT bundles, thereby optimizing the materials’ microstructure and enhancing their electrochemical performance.

Huang et al. [80] successfully synthesized CNT-Mn_3_O_4_/CoWO_4_ nanocomposites using a two-step microwave-assisted hydrothermal method, with CNTs acting as the scaffold to anchor Mn_3_O_4_ and spherical CoWO_4_ nanoparticles. The synthesis pathway is illustrated in Figure 12b. Under microwave irradiation, the “hotspots” formed on the surface of CNTs significantly enhanced the local thermal effects and electromagnetic field intensity, promoting the construction of layered heterostructures. Electrochemical testing results showed that the composite material achieves a specific capacitance of 1658.7 F/g at a current density of 1 A/g (Figure 12c). After 13,000 cycles at a high current density of 15 A/g, the initial capacitance retention is as high as 117.2%. It also features an extended voltage window of 1.15 V. Based on the constructed CNT-Mn_3_O_4_/CoWO_4_//N-C BSH device (assembly principle shown in Figure 12d), the material exhibits a maximum energy density of 67.5 Wh/kg at a power density of 1025 W/kg.

#### 2.3.3. Multicomponent Carbon-Based 3d Transition Metal Oxide Electrode Materials

Multicomponent carbon-based 3d transition metal oxide electrode materials are composites formed by combining various carbon materials (such as graphene, carbon nanotubes, activated carbon, etc.) with 3d transition metal oxides, resulting in composites with a three-dimensional structure. These materials combine the high conductivity, large surface area, and excellent stability of carbon materials with the rich electrochemical active sites of 3d transition metal oxides. By constructing porous or layered structures, they enable efficient ion transport and excellent mechanical stability, thereby optimizing the overall performance of the electrodes. Furthermore, multicomponent carbon-based 3d transition metal oxide composites synthesized via microwave-assisted methods offer enhanced electrochemical performance, providing a promising direction for the development of high-performance energy storage devices.

For example, Yetiman et al. [81] synthesized Co_3_O_4_-based nanocomposites (Co_3_O_4_@rGO@CDs) doped with carbon dots (CDs) and reduced graphene oxide (rGO) using a microwave-assisted method. The resulting electrode exhibited a specific capacitance of 936 F/g at a current density of 0.5 A/g, significantly outperforming both pristine Co_3_O_4_ (448 F/g) and Co_3_O_4_@rGO electrodes (482 F/g). Similarly, Jung et al. [82] employed microwave irradiation technology to fabricate a hierarchical NiO@srGO/CNT composite structure, which demonstrated a high specific capacitance of 1605.81 F/g at 1 A/g and maintained excellent structural stability after 10,000 charge–discharge cycles. Furthermore, Appiagyei et al. [83] utilized a microwave-assisted gel combustion method, with brown sugar as the carbon precursor, to synthesize an iron oxide–mesoporous carbon hybrid interconnected network (rGO/su-GC@Fe_2_O_3_) featuring a high surface area of 486 m^2^/g and mesoporosity. This material exhibited an impressive specific capacitance of 1978 F/g at the same current density, surpassing the performance of the NiO@srGO/CNT electrode and outperforming many other metal oxide-based electrodes and their carbon-based composites in terms of energy storage capabilities.

### 2.4. Transition Metal Oxides/3d Transition Metal Oxide Composite Materials

Carbon-based electrode materials primarily rely on the electrical double-layer mechanism for energy storage, resulting in a relatively low specific capacitance, which is mainly limited by the accessible active storage sites for ions. In contrast, transition metal oxides (TMOs) store charge through Faradaic redox reactions, exhibiting significantly higher specific capacitance. Therefore, while carbon materials demonstrate outstanding power performance, their energy density remains much lower than that of transition metal oxides.

TMOs have emerged as a research focus for supercapacitor electrode materials due to their high theoretical specific capacitance, abundant valence states, and excellent electrochemical activity [52]. When TMOs are combined with other materials, particularly 3d transition metal oxides, they can achieve significantly enhanced energy storage performance by optimizing electronic structures, strengthening interfacial interactions, improving ion transport efficiency, and increasing structural stability. This makes them ideal candidates for high-performance supercapacitor electrodes. Moreover, microwave-assisted synthesis enables the fabrication of materials with unique morphologies, such as six-petal flower-like structures, hierarchical flower-like architectures, and 3D core–shell layered structures, thereby enhancing interfacial synergistic effects. By integrating the inherent structural advantages of composite materials with the synergy induced by microwave irradiation, the electrochemical performance of these materials can be further optimized, leading to improved energy storage capability and cycling stability.

**Figure 13:**
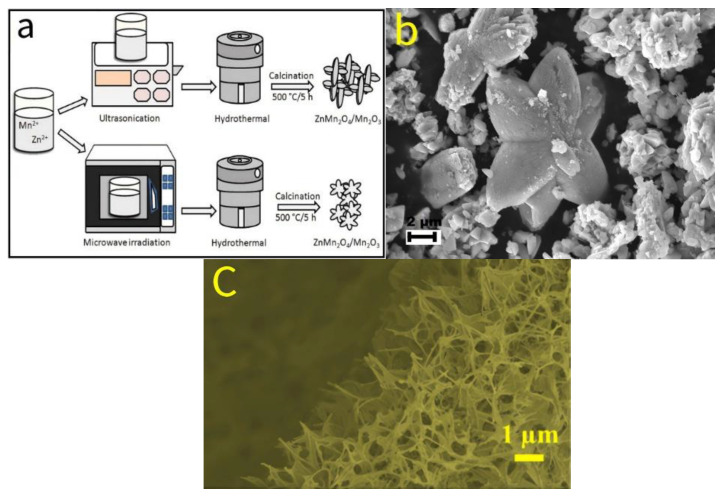
(**a**) Schematic illustration of the synthesis process of ZnMn_2_O_4_/Mn_2_O_3_ composite materials. (**b**) SEM image of ZnMn_2_O_4_/Mn_2_O_3_ composites prepared via the microwave-assisted hydrothermal method. (**c**) SEM image of core–shell nanowires@nanosheets composite structure.

Sannasi et al. [84] successfully synthesized a novel ZnMn_2_O_4_/Mn_2_O_3_ composite material via a microwave-assisted hydrothermal process, with its synthesis route illustrated in Figure 13a. Compared to the ellipsoidal/spherical structures obtained by conventional hydrothermal methods and the four-petal flower-like structures derived from ultrasound-assisted hydrothermal synthesis, the microwave-irradiated material exhibits a unique six-petal flower-like morphology (Figure 13b), demonstrating superior structural characteristics.

Similarly, Keshari et al. [85] developed a flower-like hierarchical NiO/NiMoO_4_ hybrid nanostructure using a rapid microwave-assisted synthesis approach. At a current density of 1 A/g, the specific capacitance of the microwave-treated material significantly increased from 677.8 F/g to 1147.5 F/g, representing a remarkable 69% enhancement. This improvement is primarily attributed to the synergistic effects of microwave-enhanced ex situ doped conductive carbon (50 wt%), a high specific surface area (147.9 m^2^/g), and a mesoporous structure. Furthermore, an asymmetric supercapacitor based on the NiO/NiMoO_4_//AC system achieved an energy density of 38.6 Wh/kg at a power density of 685.2 W/kg.

Additionally, Ali et al. [86] initially synthesized NiO nanowires as a core structure via a hydrothermal method and subsequently constructed a unique 3D NiO nanowires@NiO nanosheets core–shell hierarchical structure using a microwave-assisted approach (Figure 13c). In a 3 M KOH electrolyte, this electrode material exhibited an ultra-high specific capacitance of 1782 F/g at a current density of 1 A/g. The exceptional electrochemical performance is primarily attributed to the microwave-induced enhancement of the active surface area and the synergistic interactions among the composite components, thereby significantly improving the charge storage capacitance.

**Figure 14:**
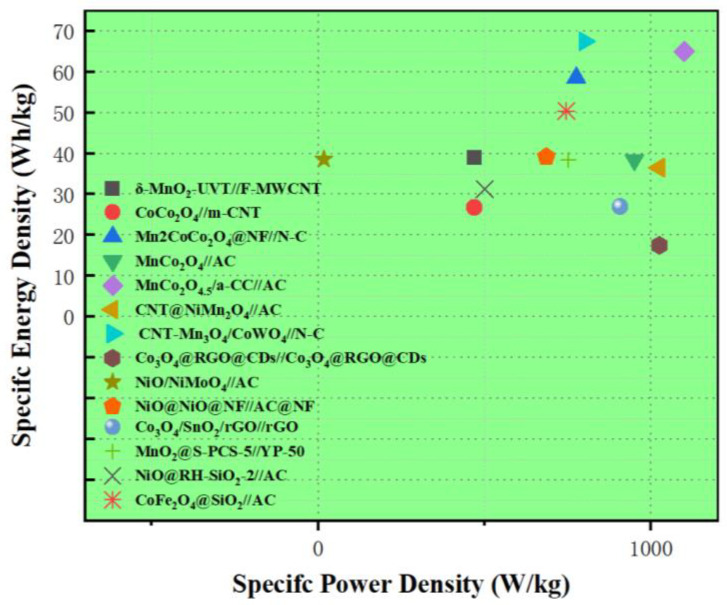
Ragone plot of all 3d transition metal oxide-based supercapacitors discussed in this work.

Due to the high theoretical specific capacitance of 3d transition metal oxides, their combination with other metal oxides often results in a higher specific capacitance compared to other 3d transition metal oxide-based electrode materials. The electrochemical performance comparison of the 3d transition metal oxide-based electrode materials discussed in this work is summarized in Table 2. Meanwhile, the corresponding Ragone plot of the various 3d transition metal oxide-based supercapacitors is presented in Figure 14.

## 3. Conclusions and Future Perspectives

### 3.1. Conclusions

In summary, this paper provides a detailed review of the research progress in microwave-assisted processes for the synthesis of 3d transition metal oxide-based nanomaterials, including 3d single-metal and binary-metal oxides, as well as their composites with carbon-based materials (such as graphene and carbon nanotubes) and other metal oxides. In the synthesis of 3d transition metal oxide-based nanomaterials, microwave technology exhibits significant advantages due to its rapid heating characteristics and unique thermal/non-thermal effects. This technique not only significantly shortens reaction times but also enables precise control over crystallinity and particle size distribution, resulting in a more uniform structure while effectively suppressing particle agglomeration. Furthermore, microwave irradiation facilitates the directional growth of nanowires and the modulation of lattice parameters, thereby enhancing material purity while simultaneously increasing specific capacitance and expanding the operating potential window. Microwave effects promote the self-assembly and morphological evolution of materials, facilitate the formation of oxygen vacancies, and optimize surface roughness and porosity. Additionally, microwave irradiation enhances interfacial synergy, allowing for precise microstructural tuning, which increases the specific surface area and the number of active sites, ultimately leading to significant improvements in overall electrochemical performance.

### 3.2. Future Perspectives

Despite significant progress in optimizing the performance of 3d transition metal oxides via microwave-assisted synthesis, several challenges remain. Future research can focus on the following areas for further improvement and advancement:

1. In-depth Investigation of Microwave-Induced Reaction Mechanisms
  Currently, the regulatory mechanisms of microwave effects in the synthesis process are not yet fully understood. Future studies should focus on elucidating the role of microwave irradiation in the structural evolution and performance modulation of materials, particularly its impact on nucleation, growth, and defect engineering.
2. Enhancement of the Practical Specific Capacitance of Electrode Materials
  The specific capacitance of microwave-synthesized 3d transition metal oxide electrode materials in practical applications remains lower than their theoretical values. Future research should prioritize the development of novel composite structures, such as multi-element doping and multi-dimensional hybrid materials, to further improve specific capacitance, energy density, and power density.
3. Advancements in In Situ Characterization Techniques
  In studies on the energy storage mechanisms of microwave-synthesized 3d transition metal oxide electrodes, conventional ex situ techniques such as XRD and XPS fail to precisely capture the dynamic changes occurring on the electrode surface during charge-discharge processes. Future research should focus on integrating in situ characterization techniques to monitor phase transitions, morphological evolution, and interfacial adsorption behavior in real-time. This approach will provide deeper insights into performance evolution mechanisms and guide the rational design of high-performance energy storage materials.

## Figures and Tables

**Table 1:** Common synthesis methods for 3d transition metal oxides and their advantages and disadvantages.

Synthesis Methods	Advantages	Disadvantages
Electrochemical deposition	Large-scale production; low cost; precise control of film thickness, deposition rate, and uniformity	Set process; required current or voltage
Chemical vapor deposition (CVD)	Good film uniformity	Expensive equipment, relatively high cost
Sol–gel method	Low cost; the prepared products have a uniform particle size distribution, good dispersion, and high purity	Easily influenced by external factors; complex reaction process; difficult to control operation
Hydrothermal/solvothermal synthesis	Convenient; adjustable reaction parameters	Low thermal energy efficiency; poor product uniformity; low yield; wall effect
Liquid-phase co-precipitation method	Easy to operate; simple process steps; high product purity; reactions are easy to control	The products are prone to aggregation, leading to poor dispersion performance
Microwave-assisted method	Economical; simple; safe; controllable; environmentally friendly; energy-saving; rapid, uniform, and selective heating; efficient; short reaction time; high yield; uniform particle size distribution and high purity of the prepared products; good reproducibility	Requires a specialized microwave reactor; most processes require solvents; difficult to commercialize

**Table 2:** Comparison of the 3d transition metal oxide-based electrode materials and their electrochemical properties discussed in this work.

Material	Material Type	Electrolyte	Specific Capacitance	Operating Voltage Window	Retention	Refs.
3d Single-Metal Oxides	δ-MnO_2_-UVT	1 M KOH	-	-	-	[15]
	MnO_2_	1 M Na_2_SO_4_	207 F/g @ 10 mV/s	-	-	[16]
	δ-MnO_2_	2 M Na_2_SO_4_	68 F/g @ 10 mV/s	-	-	[17]
	α-Fe_2_O_3_	3 M KOH	1253 F/g @ 3 mV/s	0.75 V (−0.2~0.55 V vs. Ag/AgCl)	93% (3000 cycles @ 3 mV/s)	[21]
	CoO	6 M KOH	728.8 F/g @ 1 A/g	0.85 V (−0.3~0.55 V vs. SCE)	82.3% (5000 cycles @ 15 A/g)	[26]
	CoCo_2_O_4_	6 M KOH	875 F/g @ 1 A/g	0.85 V (−0.3~0.55 V vs. SCE)	89.7% (5000 cycles @ 15 A/g)	[27]
	Mn2CoCo_2_O_4_	6 M KOH	1389 F/g @ 1 A/g	1.1 V (−0.3~0.8 V vs. SCE)	91.5% (5000 cycles @ 15 A/g)	[28]
	NiO	6 M KOH	270 F/g @ 1 A/g	-	-	[33]
	NiO	1 M KOH	87.7 F/g @ 10 mV/s	-	85% (5000 cycles @ 0.5 A/g)	[35]
3d Binary Transition Metal Oxides	NiMn_2_O_4_	6 M KOH	802 F/g @ 1 A/g	0.8 V (−0.2~0.6 V vs. SCE)	61% (5000 cycles @ 10 A/g)	[42]
	NiMnO_3_	6 M KOH	345.8 F/g @ 1 A/g	0.8 V (−0.3~0.5 V vs. SCE)	92% (1000 cycles @ 3 A/g)	[43]
	NiMn_2_O_4_	6 M KOH	768.9 F/g @ 1 A/g	0.7 V (−0.2~0.5 V vs. SCE)	85.8% (6000 cycles @ 5 A/g)	[44]
	NiFe_2_O_4_	-	-	-	-	[48]
	MnCo_2_O_4_	3 M KOH	536 F/g @ 0.5 A/g	0.5 V (0~0.5 V vs. SCE)	96% (10,000 cycles @ 5 A/g)	[59]
	MnCo_2_O_4_	2 M KOH	802 F/g @ 1 A/g	0.6 V (−0.1~0.5 V vs. SCE)	70% (1000 cycles @ 5 A/g)	[61]
	MnCo_2_O_4_	2 M KOH	1053.5 F/g @ 1 A/g	0.6 V (−0.1~0.5 V vs. SCE)	90% (2000 cycles @ 8 A/g)	[60]
	MnCo_2_O_4.5_	6 M KOH	497.8 F/g @ 1 A/g	1.05 V (0~1.05 V vs. Hg/HgO)	87.6% (5000 cycles @ 10 A/g)	[62]
Carbon-Based Materials/3d Transition Metal Oxide Composite Materials	rGO@Fe_3_O_4_	1 M KOH	771.3 F/g @ 5 mV/s	1.0 V (−0.2~0.8 V vs. Ag/AgCl)	95.1% (5000 cycles @ 0.9 A/g)	[73]
	rGO@Fe_3_O_4_	1 M KOH	471 F/g @ 10 mV/s	0.8 V (−0.2~0.6 V vs. Ag/AgCl)	96.5% (1000 cycles @ 40 mV/s)	[72]
	N-rGO@CoO	1 M KOH	744.1 F/g @ 5 mV/s	0.8 V (−0.2~0.6 V vs. Ag/AgCl)	91.3% (5000 cycles @ 70 mV/s)	[74]
	CNT@NiMn_2_O_4_	6 M KOH	915.6 F/g @ 1 A/g	0.8 V (−0.2~0.6 V vs. SCE)	93% (5000 cycles @ 5 A/g)	[78]
	Fe_3_O_4_/CNT	1 M Na_2_SO_3_	187.1 F/g @ 1 A/g	1.0 V (−1.0~0 V vs. SCE)	80.2% (1000 cycles @ 1 A/g)	[79]
	CNT-Mn_3_O_4_/CoWO_4_	-	1658.7 F/g @ 1 A/g	1.15 V (−0.2~0.95 V vs. SCE)	117.2% (13,000 cycles @ 5 A/g)	[80]
	Co_3_O_4_@rGO@CDs	2 M KOH	936 F/g @ 0.5 A/g	0.7 V (−0.1~0.6 V vs. Ag/AgCl)	88% (10,000 cycles @ 10 A/g)	[81]
	NiO@srGO/CNT	6 M KOH	1605.81 F/g @ 1 A/g	0.6 V (−0.1~0.5 V vs. SCE)	71.56% (10,000 cycles @ 1 A/g)	[82]
	rGO/su-GC@Fe_2_O_3_	2 M Na_2_SO_4_	1978 F/g @ 1 A/g	1.0 V (−0.8~0.2 V vs. Ag/AgCl)	95.2% (8000 cycles @ 10 A/g)	[83]
Transition Metal Oxides/3d Transition Metal Oxide Composite Materials	ZnMn_2_O_4_/Mn_2_O_3_	2 M KOH	380 F/g @ 0.5 A/g	0.6 V (0~0.6 V vs. Hg/HgO)	92% (2000 cycles @ 3 A/g)	[84]
	NiO/NiMoO_4_	6 M KOH	1147.5 F/g @ 1 A/g	0.5 V (0~0.5 V vs. Ag/AgCl)	-	[85]
	NiO nanowires@NiO nanosheets	3 M KOH	1782 F/g @ 1 A/g	0.6 V (0~0.6 V vs. RHE)	70.33% (7000 cycles @ 1 A/g)	[86]

## Data Availability

The original contributions presented in this study are included in the article. Further inquiries can be directed to the corresponding authors.
